# Unraveling the GM_1_ Specificity of Galectin‑1 Binding to Lipid Membranes

**DOI:** 10.1021/acsbiomedchemau.5c00040

**Published:** 2025-05-07

**Authors:** Federica Scollo, Waldemar Kulig, Gabriele Nicita, Anna-Kristin Ludwig, Joana C. Ricardo, Valeria Zito, Peter Kapusta, Ilpo Vattulainen, Marek Cebecauer, Hans-Joachim Gabius, Herbert Kaltner, Giuseppe Maccarrone, Martin Hof

**Affiliations:** † J. Heyrovský Institute of Physical Chemistry of the CAS, v. v. i., Dolejškova 2155/3, Prague 8 182 23, Czech Republic; ‡ Department of Physics, University of Helsinki, P.O. Box 64, Helsinki FI-00014, Finland; § Dipartimento di Scienze Chimiche, 9298 Università degli Studi di Catania, Viale A. Doria 6, Catania 95125, Italy; ∥ Department of Veterinary Science, Chair of Biochemistry and Chemistry, 9183 Ludwig-Maximilians-University Munich, Lena-Christ-Str.48, Planegg 82152, Germany; ⊥ Consiglio Nazionale delle Ricerche, Istituto di Cristallografia, Via P. Gaifami 18, Catania 95126, Italy

**Keywords:** galectin-1, GM_1_, GD_1_a, neuraminidase, K_d_ determination, cross-linking

## Abstract

Galectin-1 (Gal-1) is a galactose-binding protein involved in various cellular functions. Gal-1’s activity has been suggested to be connected to two molecular concepts, which are, however, lacking experimental proof: a) enhanced binding affinity of Gal-1 toward membranes containing monosialotetrahexosylganglioside (GM_1_) over disialoganglioside GD_1_a and b) cross-linking of GM_1_’s by homodimers of Gal-1. We provide evidence about the specificity and the nature of the interaction of Gal-1 with model membranes containing GM_1_ or GD_1_a, employing a broad panel of fluorescence-based and label-free experimental techniques, complemented by atomistic biomolecular simulations. Our study demonstrates that Gal-1 indeed binds specifically to GM_1_ and not to GD_1_a when embedded in membranes over a wide range of concentrations (i.e., 30 nM to 20 μM). The apparent binding constant is about tens of micromoles. On the other hand, no evidence of Gal-1/GM_1_ cross-linking was observed. Our findings suggest that cross-linking does not result from sole interactions between GM_1_ and Gal-1, indicating that in a physiological context, additional triggers are needed, which shift the GM_1_/Gal-1 equilibria toward the membrane-bound homodimeric Gal-1.

## Graphical abstract

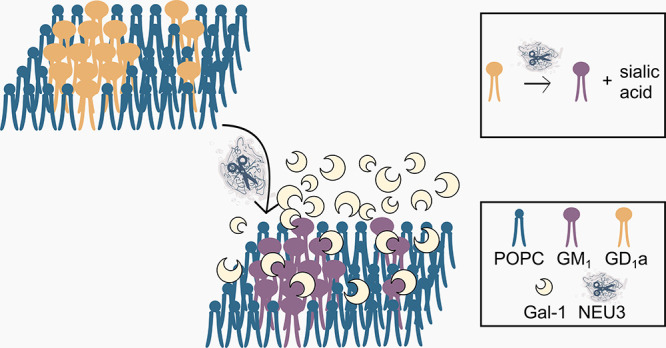

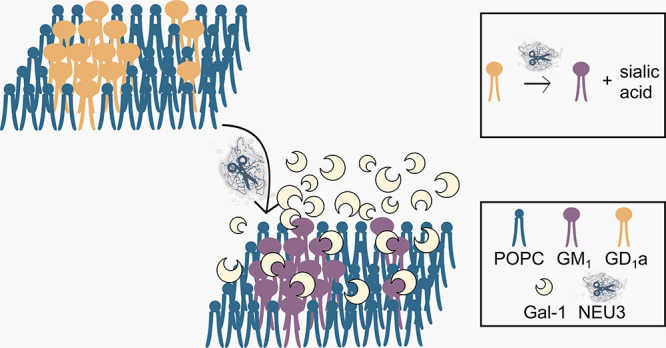

## Introduction

Galectins are a ubiquitous family of galactose-binding proteins in the nucleus, cytosol, and extracellular matrix.^1^ They are involved in various biological processes, from ribonucleic acid splicing to cell growth regulation, including cell adhesion, embryogenesis, inflammation and immune function, apoptosis, angiogenesis, and tumor metastasis.^2–15^ All galectins have a common feature: they possess one (or more) β-sandwich carbohydrate recognition domain (CRD) by which they interact with molecules having a galactoside moiety.^16^ Sixteen human galectins have been discovered,^17^ commonly classified into three structurally different types, i.e., prototype, chimera, and tandem-repeat type, based on the structural presentation of their CRD.^18^ Galectin-1 (Gal-1), which belongs to the prototype class,^19,20^ was the first of the galectins to be characterized. Nevertheless, there is still little known about its specific roles in vivo and the underlying working mechanisms.^21^ One of these uncertainties concerns its specific binding to GM_1_, one of the most abundant glycosphingolipids located at the outer layer of the plasma membrane of mainly, but not solely, neuronal cells,^22,23^ which has been postulated to be crucial for a variety of its cellular functions. The intercellular communication of regulatory and effector T cells (T_reg_ and T_eff_, respectively) might serve here as a clinically relevant example, in which the suppression of Gal-1/GM_1_ interactions has been suggested to justify imbalances in T cell communication and may causally trigger autoimmune diseases.^24^ Antigen-presenting cells (APC) activate T cell receptors of T_reg_ cells, causing upregulation of Gal-1 and its release from T_reg_ cells into the surroundings. Simultaneous activation of T_eff_ cells by APC causes an increased formation of GM_1_ through enhanced neuraminidase 3 activity, converting disialoganglioside GD_1_a to GM_1_ by removing the terminal sialic acid moiety. It has been suggested that the upregulated Gal-1 forms homodimers and cross-links to GM_1_, leading to cocross-linking with GM_1_-associated heterodimeric integrin. That cross-linking might lead to initiating a postbinding signaling cascade that activates a TRPC5 Ca^2+^ channel, which in turn blocks T_eff_ cell proliferation.^8,9,11,24–26^

This example demonstrates how Gal-1 translates the metabolic conversion of ganglioside GD_1_a to GM_1_ by neuraminidase into a specific response on the cellular level.^27^ Considering the crucial role of the specific Gal-1/GM_1_ interaction,^28^ it is essential to experimentally prove that specificity. The strongest evidence for that specificity comes from experiments in neuroblastoma cells.^19,27,29^ In these contributions, the Gal-1 binding to the cell surface is quantified by radioisotope I^125^ Gal-1 marking^19,29^ or by Gal-1 visualization via fluorescently labeled antibodies.^27^ The concept of these experiments is a) to vary GM_1_ concentrations on the cell surface by using different cell lines or interfering with the neuraminidase activity or b) blocking surface GM_1_ by antibodies or cholera toxin B subunit. These experiments indicate that the surface concentration of Gal-1 is correlated to the availability of the GM_1_ pentasaccharide headgroup. Nevertheless, these in vivo experiments do not give direct proof for Gal-1 binding to GM_1_, which gives the motivation to characterize the Gal-1/GM_1_ interaction in vitro.

Frontal affinity chromatography, or nuclear magnetic resonance (NMR), isothermal titration calorimetry (ITC), and surface plasmon resonance (SPR) experiments have been performed with the isolated GM_1_ pentasaccharide.^30–32^ The outcome of these experiments is contradictory. Moreover, as the lipid moiety was neglected, these experiments show (if at all) that the GM_1_ pentasaccharide headgroup with its terminal galactose moiety provides a docking site for Gal-1, but not that Gal-1 binds to GM_1_ when embedded in a membrane environment. To the best of our knowledge, an X-ray reflectivity and grazing incidence diffraction study on a lipid monolayer at the air/water interface^33^ and a chemically induced dynamic nuclear polarization study using GM_1_ in dodecylphosphocholine micelles^30,31^ are the only in vitro studies using GM_1_. Of note, significant efforts were made using supramolecular self-assembled polymers with controlled size and high glycan surface concentration to investigate galectin avidity.^34,35^ However, these studies do not provide insight into the GM_1_ specificity since they are based on lactose glycodendrimers. Altogether, these in vitro studies do not yield convincing proof for a specific Gal-1/GM_1_ interaction.

Moreover, it has been suggested that GM_1_ cross-linking by homodimer Gal-1 might lead to 2-dimensional glycan-galectin aggregates, called Gal-1 “lattices”.^15,28,36,37^ Gal-1 and Gal-1 “lattice” are claimed to play a crucial role in glycocalyx organization and regulation.^38^ However, the literature does not provide any direct proof of GM_1_ being involved in Gal-1 cross-linking or surface aggregation. The apparent imbalance between the suggested cellular role of the Gal-1/GM_1_ interaction and the lack of experimental data calls for a thorough characterization of the Gal-1 interactions with GM_1_ embedded in well-controlled membrane model systems.

In this work, we investigate the interaction of wild-type Gal-1 with model membranes containing GM_1_ or GD_1_a, combining fluorescence-based and label-free techniques. Specifically, we employed Förster resonance energy transfer (FRET), confocal fluorescence microscopy (FM), quartz crystal microbalance with dissipation monitoring (QCM-D), and ITC techniques. The experiments are complemented by all-atom molecular dynamics (MD) simulations to gain insight into the Gal-1/GM_1_ specificity at a molecular level. All of these techniques provide clear evidence for the GM_1_ specificity of Gal-1 binding to lipid membranes characterized by micromolar binding constants.

## Experimental Procedures

### Materials

1-palmitoyl-2-oleoyl-*sn*-glycero-3-phosphocholine (POPC), Ganglioside GM_1_ (Ovine Brain), 1,2-dioleoyl-*sn*-glycero-3-phosphoethanolamine-N- (cap biotinyl) (DOPE-cap-biotin), and 1,2-dioleoyl-*sn*-glycero-3-phosphoethanolamine-*N*-(lissamine rhodamine B sulfonyl) (ammonium salt) were purchased from Avanti Polar Lipid (Alabaster, AL, USA). Ganglioside GD_1_a disodium salt (bovine brain) was purchased from Enzo Life Sciences (Farmingdale, NY, USA). Chloroform (containing amylenes as a stabilizer, ACS reagent, ≥99.8%), methanol (ACS reagent, ≥99.8%), phosphate buffered saline (PBS) (tablets), 4-(2-Hydroxyethyl)-piperazine-1-ethanesulfonic acid sodium salt (HEPES), sodium chloride (NaCl), hydrogen peroxide Solution (30%), ammonia (25%), sucrose, bovine serum albumin (BSA), β-1-thio-d-galactopyranoside (IPTG), lactose monohydrate, iodoacetamide, sodium dodecyl sulfate, 2-ammino-2-(idrossimetil)-1,3-propandiolo (Trizma base), and ammonium peroxodisulfate were acquired from Sigma-Aldrich (St. Louis, MO, USA) (all 99% purity, unless otherwise stated). DOPE (1,2-Dioleoyl-*sn*-glycero-3-phosphoethanolamine) labeled with Atto-633 was provided by Atto-Tec GmbH (Siegen, Germany). TAMRA maleimide 6-isomer was purchased from Lumiprobe (Hannover, Germany). Terrific Broth (TB) medium (TB), potassium dihydrogen phosphate, dipotassium hydrogen phosphate, Luria Broth medium, sodium bicarbonate, ROTIPHORESENF-Acrylamid/Bis-Solution 30 (29:1), and tetramethylethylendiamin were purchased from Roth (Karlsruhe, Germany), while PD10 and columns Sepharose 4B (for the self-made lactosylated Sepharose 4B) were from Cytiva (Freiburg, Germany). Biotinylated bovine serum albumin, cholera toxin subunit B (recombinant), Alexa Fluor 488 conjugate, and Alexa Fluor 532 NHS ester (succinimidyl ester) were purchased from Thermo Fisher (Waltham, MA, USA). Rhodamine B was purchased from Molecular Probes (Eugene, OR, USA), and streptavidin was purchased from IBA Life Sciences (Göttingen, Germany). Escherichia coli BL21 (DE3) pLysS cells were provided by Promega (Walldorf, Germany).

### Recombinant Protein Expression and Purification

For recombinant protein expression of wild-type human Gal-1, E. coli BL21 (DE3) pLysS cells were transformed with the pGEMEX-Gal-1 plasmid. Transformed bacteria were grown for 16 h at 37 °C in Luria Broth medium containing the appropriate selected antibiotic. For protein expression, medium was changed to TB medium (TB). After initial growth for 2 – 3 h at 37 °C in TB medium up to an OD600 nm of 0.6–0.8, gene expression was induced using 100 μM β-1-thio-d-galactopyranoside (IPTG), and bacteria were cultured at 37 °C for an additional 16 h. Cells were harvested and washed, and bacteria pellets were frozen for 2 h at −20 °C before being lysed by sonication at 4 °C. The protein was purified from the bacterial extracts after lysis by affinity chromatography on lactosylated Sepharose 4B. The lactosylated Sepharose 4B column-bound protein was labeled directly with a four-molar excess of maleimide-TAMRA (TMR) dye, followed by extensive washing steps before the elution. Therefore, the buffer was changed to labeling buffer (0.1 M sodium bicarbonate, pH = 8.3), and labeling occurred according to the manufacturer’s instructions, rotating overnight at 4 °C in the dark. After labeling, free dye was washed out of the column with 20 mM PBS pH = 7.2 buffer, and active protein was eluted with 50 mM lactose in 20 mM PBS pH = 7.2, followed by buffer exchange to 10 mM PBS pH = 7.2 via a PD10 column to remove lactose. Purity was ascertained by one-dimensional gel electrophoresis under denaturing conditions.

### Model Membranes Preparation

Briefly, the desired lipid composition vesicles, large unilamellar vesicles (LUVs) or giant unilamellar vesicles (GUVs), were obtained from the chloroform stock solutions using extrusion or electroformation methods.^39–41^ 1% of the DOPE-Atto633 probe or 1% of DPPE-cap-biotin were added to the chloroform solution when needed. The mixture was dried under nitrogen flow in a test tube (LUVs) or spread onto two preozonized and preheated titanium plates (GUVs), in both cases further evaporated under a vacuum for at least 3 h. The dry lipid films were hydrated and vortexed in a buffer solution (10 mM PBS, 137 mM NaCl, 0.27 mM KCl, pH 7.4) for LUV preparation. The lipid-coated plates were assembled and filled with sucrose in water (107 mOsm kg^–1^) and then placed on a heating plate at approximately 45 °C. Before the LUVs extrusion, seven cycles of freeze and thaw were performed using liquid nitrogen and a water bath set above the phase transition temperature characteristic of the lipid used (42–45 °C). The dispersion was later extruded (101 times at 45 °C) through polycarbonate filters (pore diameter 100 nm, Nuclepore, Pleasanton, CA, USA) mounted in a miniextruder (Avanti Polar Lipid, Birmingham, England) fitted with two 0.5 mL Hamilton syringes (Hamilton, Reno, NV). For GUV preparation, the voltage was increased stepwise from 0.250 to 3.5 V (peak-to-peak voltage) for 65 min and then kept at 3.5 V for 1 h. In the final step, the frequency was decreased stepwise from 10 to 4 Hz, and the voltage was kept at 3.5 V for 1 h to detach the formed liposomes. The temperature was kept at 45 °C. The obtained GUV dispersions were later diluted with buffer of the same osmolality (10 mM HEPES, 50 mM NaCl, pH = 7.4, 107 mOsm kg^–1^).

### Fluorescence Confocal Microscopy and Fluorescence Correlation Spectroscopy

Prior to imaging acquisition, the μ-Slide 8 well-ibidi chamber (Grafelfing, Germany) chamber with a glass bottom was coated with 200 μL of BSA-biot (0.1 mg/mL), 200 μL of streptavidin (2 μg/mL), waiting 30 min each step, and washing all the wells with mQ water after each step of coating. In each well, we added 80 μL of GUVs (HEPES 10 mM, NaCl 50 mM, pH = 7.4, 107 mOsm kg^–1^) and after letting the GUVs attach to the coated bottom for 30 min, 30 nM of Gal-1/TMR was added and diluted to reach a total volume of 450 μL (total lipids concentration of about 20 μM). Prior to each measurement, the background, i.e., the unlabeled GUVs, was checked. For Fluorescence Correlation Spectroscopy (FCS) experiments, to avoid Gal-1/TMR adsorbing on the glass bottom, we coated the chambers using only BSA. Image acquisition and FCS measurements were performed on a home-built confocal microscope. Pulsed diode laser (532 nm) was used at a 25 MHz repetition rate. A quad-band dichroic mirror (375/470/532/640) was used to upreflect the light onto a water immersion objective (60×, NA 1.2). Furthermore, a 570–620 nm bandpass filter was employed. For imaging acquisition, the power of the laser was kept below 5 μW (measured at the end of the fiber), and each image was recorded at a different resolution depending on the size of the single GUV, scanning in the monodirectional mode. The images were acquired after 1 h from the addition of the protein to the GUVs chamber. The experiments were performed twice, with two different sets of electroformed GUVs (two biological replicates; overall > 10 GUVs were analyzed). For FCS, the power of the laser was set to 10 μW (measured at the end of the fiber), and each point was acquired for 2 min.

### Förster Resonance Energy Transfer

Time-resolved measurements were performed by using a modular FluoTime250 spectrofluorometer (PicoQuant GmbH, Berlin, Germany) using time-correlated single photon counting. The setup has been equipped with a green excitation laser (PicoQuant LDH-D-TA 532, pulse width less than 100 ps, 80 MHz maximum repetition rate, emission peaking at 531 nm) and an HPMA-06 hybrid photomultiplier tube. The overall instrument response function width was around 120 ps fwhm. An emission long-pass cutoff filter (540 nm) was used to eliminate the scattered excitation light. The sample was measured at a 578 nm emission wavelength. The monochromator slit width was adjusted according to the protein emission intensity and then kept constant during the whole titration. Titrations were performed by adding different volumes of red-labeled LUVs of different compositions (4 mM) to 0.5 μM of Gal-1/TMR into an Ultra-Micro Cell cuvette (Hellma, Merck KGaA, Darmstadt, Germany). After 10 min from each addition, a decay curve was recorded with the setup described above. The so-obtained set of decays was analyzed globally, using iterative reconvolution fitting of a three-exponential function using EasyTau 2 Software (PicoQuant GmbH, Berlin, Germany). The Amplitude-Weighted Average Lifetime has been calculated and plotted (together with its standard deviation, n = 5, four different extrusions for POPC and POPC/GM_1_ data, n = 2, two different extrusions for POPC/GD_1_a data).

### Quartz Crystal Microbalance with Dissipation Monitoring

The measurements were conducted using a Quartz Crystal Microbalance with a Dissipation monitoring system, equipped with a quartz crystal of 5 MHz (diameter 14 mm) (Novaetech Srl, Pompei, Italy). Before each measurement, the sensor had to be properly cleaned and activated as previously described.^42,43^ Briefly, a solution of 5:1:1 of distilled water, ammonia solution (25%) and hydrogen peroxide (30%) was used to treat the sensor at 70 °C for 20 min. Afterward, the sensor was rinsed with water, dried under nitrogen flow, and then exposed under a UV lamp for 10 min, twice. Before performing each measurement, the sensor was calibrated, and then both the frequency and dissipation changes at the first overtone were monitored previously in the air and then following the injection of the buffer. Once the frequency and the dissipation were stable, 1 mM of lipid dispersion of the desired composition was injected into the chamber until further stabilization. In the case of POPC/GM_1_ and POPC/GD_1_a, the observed frequency shifts were smaller than the ones related to POPC depositions, due to an incomplete coverage of the sensor. Therefore, after the deposition of the vesicles containing gangliosides, the inert dispersion of POPC was perfused until saturation of the sensor, corresponding to stabilization of the frequency value. Once the frequency reached a constant value, the Gal-1 solution (10 mM PBS, 137 mM NaCl, 0.27 mM KCl, pH 7.4) was introduced into the chamber, succeeded once again by the buffer. Each injection step was followed again by a buffer perfusion. A flow rate of 20 μL/min was used and kept constant during the whole experiment.

### Isothermal Titration Calorimetry

The experiments were performed by using an ITC Nano Active Control Calorimeter (TA Instruments, New Castle, DE, USA) and Auto-iTC200 (Malvern Panalytical, Malvern, United Kingdom). The first one was equipped with a gold cell (volume of 988 μL, provided by the manufacturer), whereas the second was equipped with a coin-shaped Hastelloy cell (200 μL). Three syringes, 40, 100, and 250 μL volumes equipped with a shaker, have been used to perform the titrations. Each solution used was previously degassed for 20 min. This time was optimized to minimize the variations in the concentrations of the prepared solutions. The reliability of the results obtained with the ITC Nano Active Control Calorimeter was further strengthened by the precision and accuracy of the calorimetric apparatus and thoroughly tested by using a chemical calibration procedure^44^ rather than the simple electrical calibration recommended by the ITC manufacturers.^45,46^ The concentrations used in the experiments are within the ranges 2–10 μM and 1.5 mM for Gal-1 and LUVs, respectively. All of the experiments were carried out in overfill mode at 25 °C, in a temperature-controlled room (25.0 ± 0.4 °C), and under stirring (750 rpm).

### Molecular Dynamics Simulations Setup

MD simulations were carried out on five protein–lipid bilayer systems, whose detailed molecular compositions are given in Table 2. In each of these systems, the homodimeric Gal-1 (PDB ID: 1GZW) was placed at least 2 nm above the surface of the lipid bilayer in three random orientations. An appropriate number of water molecules was added to solvate the system fully. We added 0.15 M KCl and inserted additional potassium ions to neutralize the net charge of the system. All molecules in the system were described using the CHARMM36m force field.^47^ The resulting 15 MD simulations were energy minimized, and 1 μs long simulations was carried out with a time step of 2 fs. The first 500 ns of the simulation time were considered as equilibration time and excluded from subsequent analyses. The periodic boundary conditions were used in all three dimensions. The simulations were performed in the NpT ensemble with a temperature of 298 K kept using the Nosé-Hoover thermostat^48,49^ and a pressure of 1 atm kept by the Parrinello–Rahman barostat.^50^ The coupling constants for temperature and pressure were 1 and 5 ps, respectively. Temperature coupling for protein, lipid bilayer, and solvent (water and ions) was independent. The pressure in the membrane plane (*xy* plane) was maintained independently from pressure along the bilayer normal (semi-isotropic pressure coupling). All simulations were performed using GROMACS 2020.5 software.^51^

**Table 1:** Lipid Compositions Used in This Study

name	POPC	GM_1_	GD_1_a
POPC	100	-	-
POPC/GM_1_	96	4	
POPC/GD_1_a	96	-	4

**Table 2:** Molecular Compositions of the Systems Used in the MD Simulations

label	Gal-1 homodimer	POPC	GM_1_	GD_1_a	K^+^	Cl^–^	water	number of replicates	simulation time per replicate (μs)
POPC	1	256	-	-	28	22	32,800	3	1
POPC/GM_1_ (4 mol %)	1	246	10	-	68	52	41,541	3	1
POPC/GM_1_ (10 mol %)^a^	1	230	26	-	80	48	40,254	3	1
POPC/GD_1_a (4 mol %)	1	246	-	10	83	57	43,150	3	1
POPC/GD_1_a (10 mol %)^a^	1	230	-	26	114	56	42,846	3	1

a A 10 mol % GM_1_ has been used in the MD simulations to enhance the sampling.

The calculation of the potential of mean force (PMF) between the Gal-1 monomers in water was carried out using the umbrella sampling technique.^52,53^ The Gal-1 homodimer was placed in the middle of the cubic simulation box. The system was solvated, and potassium ions were added to neutralize the net charge of the system. Before the beginning of the pulling simulation, the system was energy minimized and equilibrated for 200 ns. Thirty-four windows with 0.1 nm spacing were constructed by pulling apart the centers of mass of the Gal-1 monomers. The pulling rate of 0.2 nm/ns with a 2000 kJ mol^–1^ nm^–2^ force constant was used. Each window was simulated for 200 ns, with the first 100 ns considered as equilibration time and removed from the free energy calculations. The PMF was calculated using the weighted histogram analysis method implemented in GROMACS.^54^ Errors were estimated using the Bayesian bootstrapping method.^55^

## Results and Discussion

On a cell surface, glycosphingolipids are prominently encountered and tightly controlled. Shifts in ganglioside profiles occur in cell activation/differentiation or neuronal regeneration. The conversion of ganglioside GD_1_a to GM_1_ via enzymatic desialylation by the plasma membrane neuraminidase 3 has been suggested to be read by a concerted upregulation of Gal-1, with the ensuing growth control of neuroblastoma cells, axon regeneration, and effector/regulatory T cell communication in autoimmunity control.^28^ This fascinating concept is based on the elevated binding affinity of Gal-1 toward GM_1_ over GD_1_a when embedded in membranes, an assumption which, however, lacks experimental proof.

To characterize the lipid specificity of Gal-1 binding, phospholipid bilayers are an ideal model system. More specifically, we monitored Gal-1 binding to diverse unilamellar vesicles composed of palmitoyl-oleoylphosphatidylcholine (POPC) with and without GM_1_ or GD_1_a in relevant concentrations of 4 mol %, the highest reported levels of GM_1_ in neurons.^28,56,57^ At these ganglioside concentrations, GM_1_ and GD_1_a organize into transient and fluid nanodomains.^58,59^ First, we used unlabeled GUVs containing POPC with 4 mol % of GM_1_ to visualize their interaction with Gal-1 labeled with tetramethylrhodamine dye (TMR), referred to here as Gal-1/TMR (for detailed characterization see Supporting Information (SI)). The GUVs were visualized by using confocal microscopy. Single-channel detection revealed a fluorescence emission on the GUVs in the spectral emission range of the TMR, indicating that Gal-1/TMR localizes and accumulates on the POPC/GM_1_ GUVs, unlike on the POPC or POPC/GD_1_a GUVs (as shown in Figure 1a). FM data are summarized in Figure 1b and show that Gal-1/TMR adsorbs onto (84 ± 6) % of POPC/GM_1_ GUVs membranes and only onto (15 ± 5) % of POPC GUVs and (11 ± 6) % of POPC/GD_1_a GUVs, suggesting a specific interaction between Gal-1 and GM_1_.

**Figure 1:**
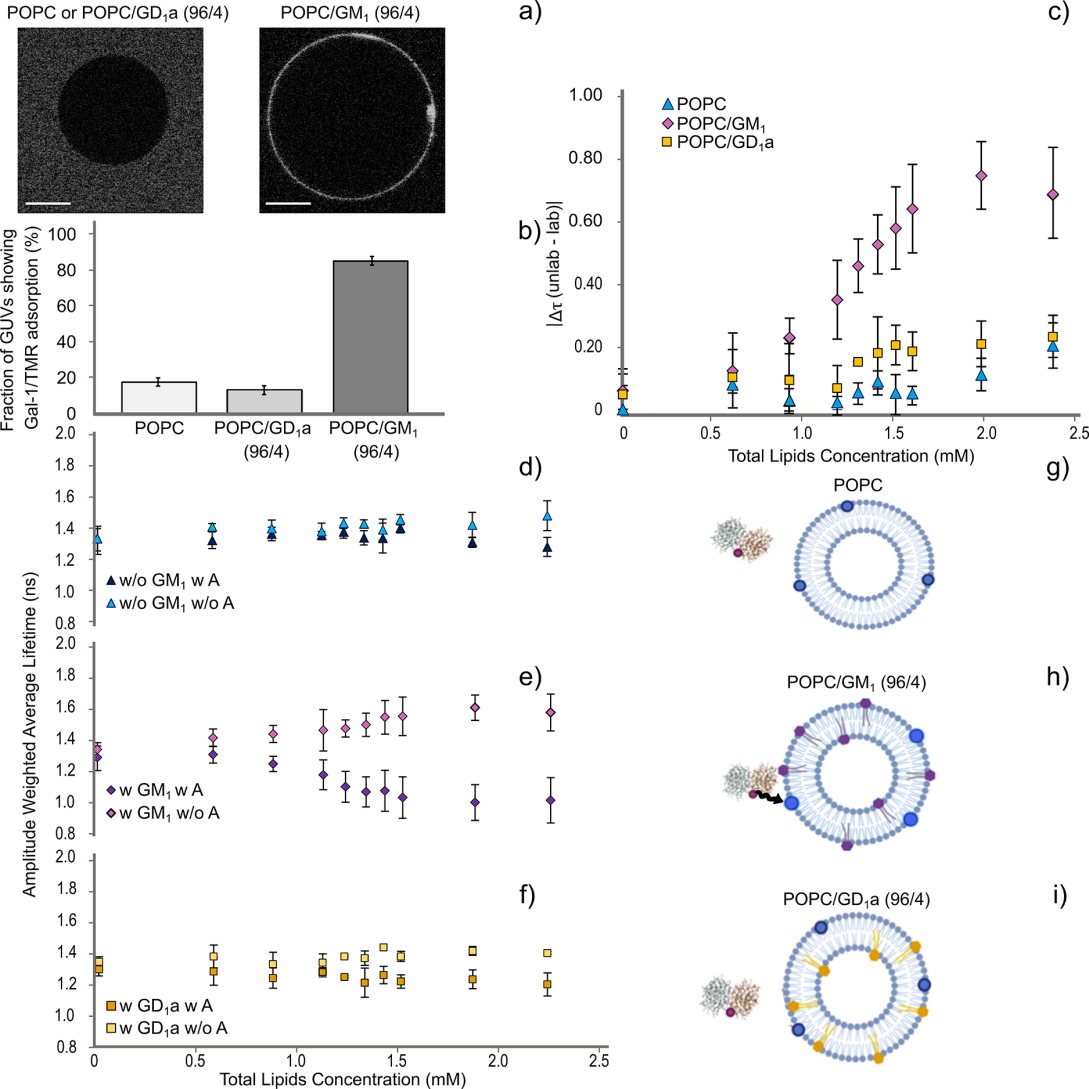
Representative confocal images of Gal-1/TMR with unlabeled GUVs and FRET data measured on Gal-1/TMR and labeled and unlabeled LUVs. (a) Representative *xy* cross sections of unlabeled POPC or POPC/GD_1_a and POPC/GM_1_ incubated with Gal-1/TMR (30 nM). Scale bars are shown on the bottom left (10 μm). b) Fraction of GUVs showing adsorption and associated error of Gal-1/TMR at the membrane of POPC, POPC/GD_1_a, and POPC/GM_1_ (see Table 1 for the details). *n* > 15 (at least three different electroformations). (c) |Δτ (unlab – lab)| as a function of total lipid concentration for three different compositions, i.e., POPC (blue triangles), POPC/GM_1_ (purple rhombs), and POPC/GD_1_a (yellow squares). |Δτ (unlab - lab)| is defined as the absolute value of the differences between the amplitude-weighted average lifetime of the Gal-1/TMR (the donor, 500 nM) in the presence of LUVs noncontaining the acceptor (labeled as A) and the same in the presence of LUVs containing the acceptor (1% of DOPE-Atto 633). Error bars represent the SEM. (d–f) Amplitude weighted average lifetime of Gal-1/TMR as a function of increasing dispersion concentration for POPC, POPC/GM_1_, and POPC/GD_1_a, respectively. All the decay curves were acquired at 25 °C. Error bars represent the SD (*n* = 5 for POPC and POPC/GM_1_ and *n* = 2 for POPC/GD_1_a). The average was calculated on samples prepared by at least two different extrusions. (g, h, i) Illustration of Gal-1/TMR and POPC, POPC/GM_1_, and POPC/GD_1_a LUVs, respectively. The structure of the protein was taken from PDB (https://www.rcsb.org/3d-view/1SLA). TMR and DOPE-Atto 633 dyes are shown in the illustration as red dots attached to the protein and blue dots intercalated in the vesicles, respectively. POPC, GM_1_, and GD_1_a vesicles were done in Biorender Software and represented as light blue, purple, and yellow lipids, respectively. The black arrow shows the occurrence of the energy transfer from the donor (Gal-1/TMR) to the acceptor (POPC/GM_1_ + DOPE-Atto 633).

To better decipher this specificity, we performed FRET experiments. Herein, we focused on the fluorescence lifetime of the donor (Gal-1/TMR) and monitored lifetime changes upon titration of LUVs composed of POPC, POPC/GM_1_, or POPC/GD_1_a into the galectin-containing solution using 1% of DOPE-Atto 633 in these lipid mixtures as a FRET acceptor. Figure 1g, h, i depicts the illustration of the systems used. These data show that the lifetime of the protein remains constant when adding labeled and unlabeled POPC as well as POPC/GD_1_a (Figure 1d, f). However, the lifetime of Gal-1/TMR in the presence of POPC/GM_1_ changes significantly (Figure 1e). Specifically, titrating with LUVs embedded with the acceptor causes a decrease in the donor lifetime, thus indicating the occurrence of FRET (Figure 1e).

Conversely, we observed an increase in the TMR fluorescence lifetime in the absence of the acceptor (Figure 1e). This can be explained by different nanoenvironments sensed by the dye linked to Gal-1 due to the change from bulk to the membrane-bound state. For a better visualization of the data, we plotted the absolute difference in Gal-1/TMR lifetime in the absence and in the presence of the acceptor-containing vesicles against the total concentration of phospholipids (Δτ (unlab – lab)—Figure 1c). These data (Figure 1c–f) show that the presence of GM_1_ in the bilayer affects the Gal-1/TMR’s lifetime. This effect is specific to GM_1_ and not to its precursor GD_1_a.

The titration curves in Figure 1e show a threshold lipid concentration of > 0.6 mM for a detectable change in the fluorescence time. Apparently, the binding of fluorescently labeled Gal-1 is only detectable at such elevated lipid concentrations. As shown in the Supporting Information (Figure S3b), UV–vis characterization of the Gal-1/TMR indicates that about 55% of the protein is unlabeled and 45% of the Gal-1 molecules are labeled with one or more fluorophores. We suggest that unlabeled Gal-1 predominately binds at lipid concentrations lower than 0.6 mM, which trivially cannot be detected by fluorescence. Thus, the apparent threshold lipid concentration of 0.6 mM for the change of the fluorescence lifetime is a consequence of a significant decrease of Gal-1 binding affinity due to TMR labeling. It is indeed generally known that labeling might decrease the binding affinities of proteins.^60–62^ In summary, fluorescence experiments demonstrate the specificity of the interaction of Gal-1 with GM_1_ or GD_1_a-containing membranes. However, the covalent attachment of the TMR fluorescent dye apparently decreases the binding affinity, a finding which should be kept in mind when using fluorescence-based techniques when characterizing the binding of galectins to membranes.

Next, we quantified the binding of unlabeled Gal-1 to LUVs using label-free techniques, specifically QCM-D and ITC. QCM-D is a surface technique that quantifies the material deposited onto the sensor with a sensitivity of the order of ng/cm^2^.^63^ In each experiment, we monitored frequency changes induced by the deposition of intact LUVs (Figure 2b, step I; see the Supporting Information for the details) and after the injection of the protein (Figure 2b, step III). The presence of 4 mol % of GM_1_ or GD_1_a in the vesicles introduces negative charges, which are not present in the homogeneous POPC vesicles. This leads to more significant electrostatic repulsive interactions between the adsorbed vesicles and prevents complete coverage of the gold sensor. To ensure consistent coverage of the sensor in experiments with different lipid compositions, a second step of surface coating with uncharged POPC lipid vesicles (Figure 2b, step II) was introduced (see Supporting Information, Table S1 and Figure 2b for a schematic representation). A representative frequency plot for each lipid composition is presented in Figure 2a. To gain insight into the binding of Gal-1 to lipid membranes, we focused on the frequency shifts due to the injection of 10 μM Gal-1 (Figure 2a and b, step III). This frequency is obtained by calculating the difference between the final frequency reached after the washing step and the initial frequency that was observed prior to the addition of the protein (see Supporting Information for details). The results are presented in Figure 2c as a bar plot. These experiments yielded the following observations: a) a small increase in frequency was noted for POPC (Δ*f* = −6 Hz ± 2 Hz), probably attributed to the slow detachment of vesicles from the sensor; b) Gal-1 did not cause a significant change in frequency to the previously deposited layer of POPC/GD_1_a vesicles; c) intriguingly, the addition of Gal-1 to POPC/GM_1_ LUVs led to a significant decrease in the frequency (Δ*f* = 25 Hz ± 2 Hz), providing evidence that Gal-1 binds to the POPC/GM_1_ LUVs, consistent with findings from fluorescence-based experiments (Figure 1). The fraction of protein bound was calculated by dividing each frequency shift related to a certain concentration of the Gal-1 by the highest frequency shift observed (i.e., 25 Hz at 10 μM of Gal-1). The results are reported in Figure 2d, illustrating the fraction bound as a function of different concentrations of Gal-1. The curve was then fitted, yielding an apparent *K*_d_ of (4 ± 1) μM. Of note, we deposited intact POPC LUVs containing physiologically relevant concentrations of GM_1_ instead of forming supported lipid bilayers and thus avoided changing the curvature of the bilayer, which could affect its physical-chemical properties. The apparent *K*_d_ is in the same range as estimated by radioisotope I^125^ Gal-1 marking experiments to neuroblastoma cells.^19,27,29^

**Figure 2:**
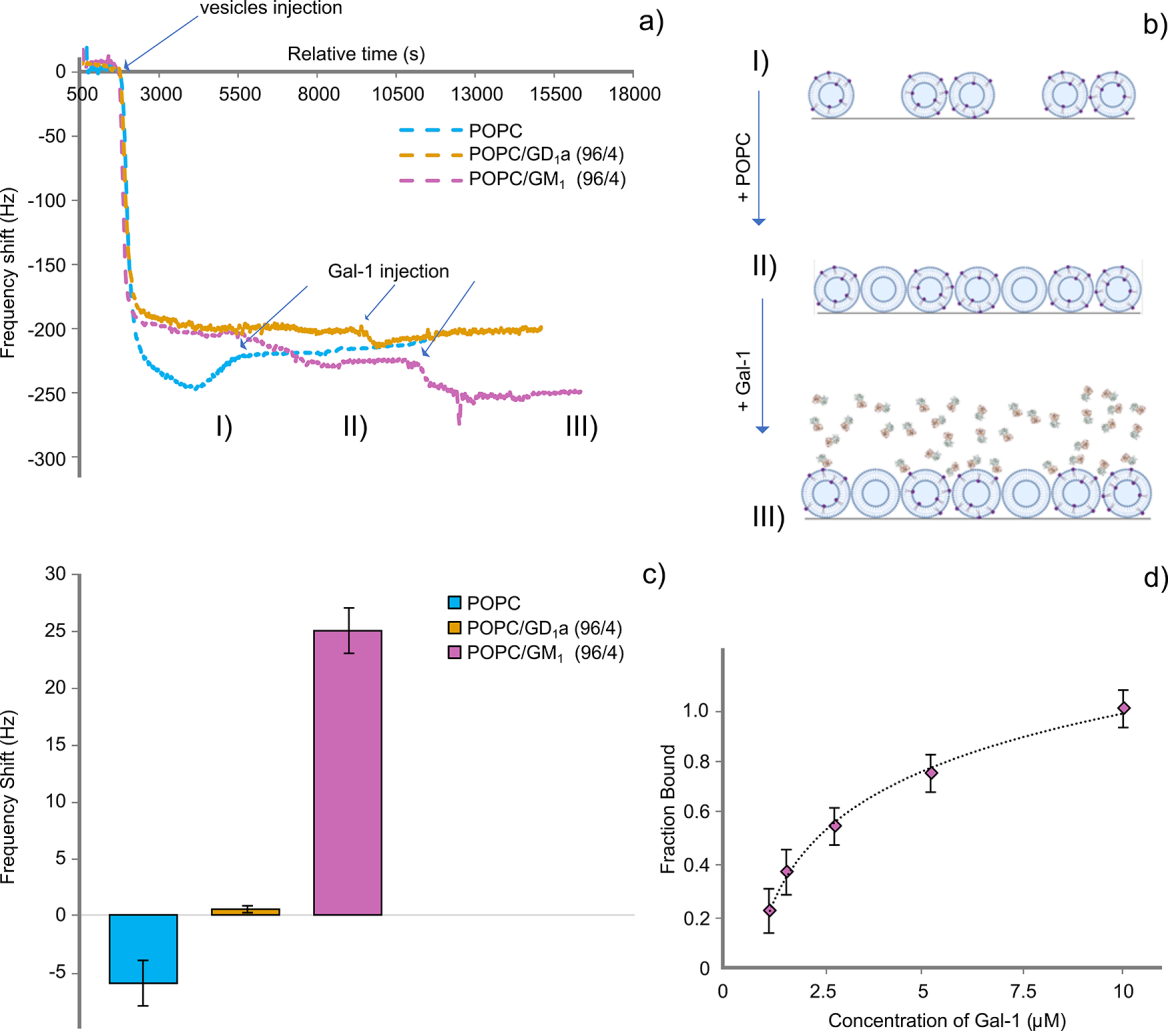
QCM-D data. (a) Representative frequency plots for POPC, POPC/GM_1_, and POPC/GD_1_a vesicle deposition on the QCM-D sensor, followed by injecting additional POPC (if needed) and Gal-1. (b) Schematic illustration representing the three steps of the experiment flow. (I) injection of the lipid vesicle of interest (1 mM total lipid concentration) (II) injection of additional POPC (1 mM total lipid concentration) in case of GM_1_/GD_1_a containing vesicles and (III) injection of the Gal-1 (10 μM). (c) Δ*f* of the III step calculated as frequency after–before Gal-1 injection for three POPC (blue bar), POPC/GM_1_ (purple bar), and POPC/GD_1_a (yellow bar). Error bars signify the SEM (*n* = 3 for POPC/GM_1_ and *n* = 2 for POPC and POPC/GD_1_a). (d) Fraction of the Gal-1 to POPC/GM_1_ vesicles as a function of Gal-1 concentration. The fraction bound was calculated by dividing each frequency shift by the maximum frequency shift achieved at the saturation point, thus the maximum amount of protein that can bind to the supported lipid vesicles. The same experiment flow was adopted in (a–d) (described in b) but varying the concentration of the Gal-1 at the third step. The formula used to fit the data is: FB = $\frac{c\times {K_{d}}^{-1}}{1+c\times {K_{d}}^{-1}}$ , where FB is the fraction bound, *c* is the concentration of the protein adsorbed, and *K*_d_ is the dissociation constant.^64^ For each concentration, the experiment was repeated in triplicates. Error bars represent the SEM (*n* = 3). All the data are acquired at 25–30 °C.

Nevertheless, *K*_d_s determined by surface-based methods as QCM-D can deviate from solution methods for several reasons, i.e., chemical heterogeneity due to the immobilization procedure, crowding/steric hindrance, or restriction in the degree of freedom or diffusional properties.^65,66^ As this can modify the thermodynamics of the interaction, we employed ITC to gain detailed insight into the thermodynamics of the Gal-1/GM_1_ interaction under equilibrium conditions without vesicle immobilization.^67^ Titrating the Gal-1 protein in a buffer with the dispersion of POPC/GM_1_ LUVs enabled us to determine the heat released or absorbed by the protein–lipid membrane interaction (Figure 3a).

**Figure 3:**
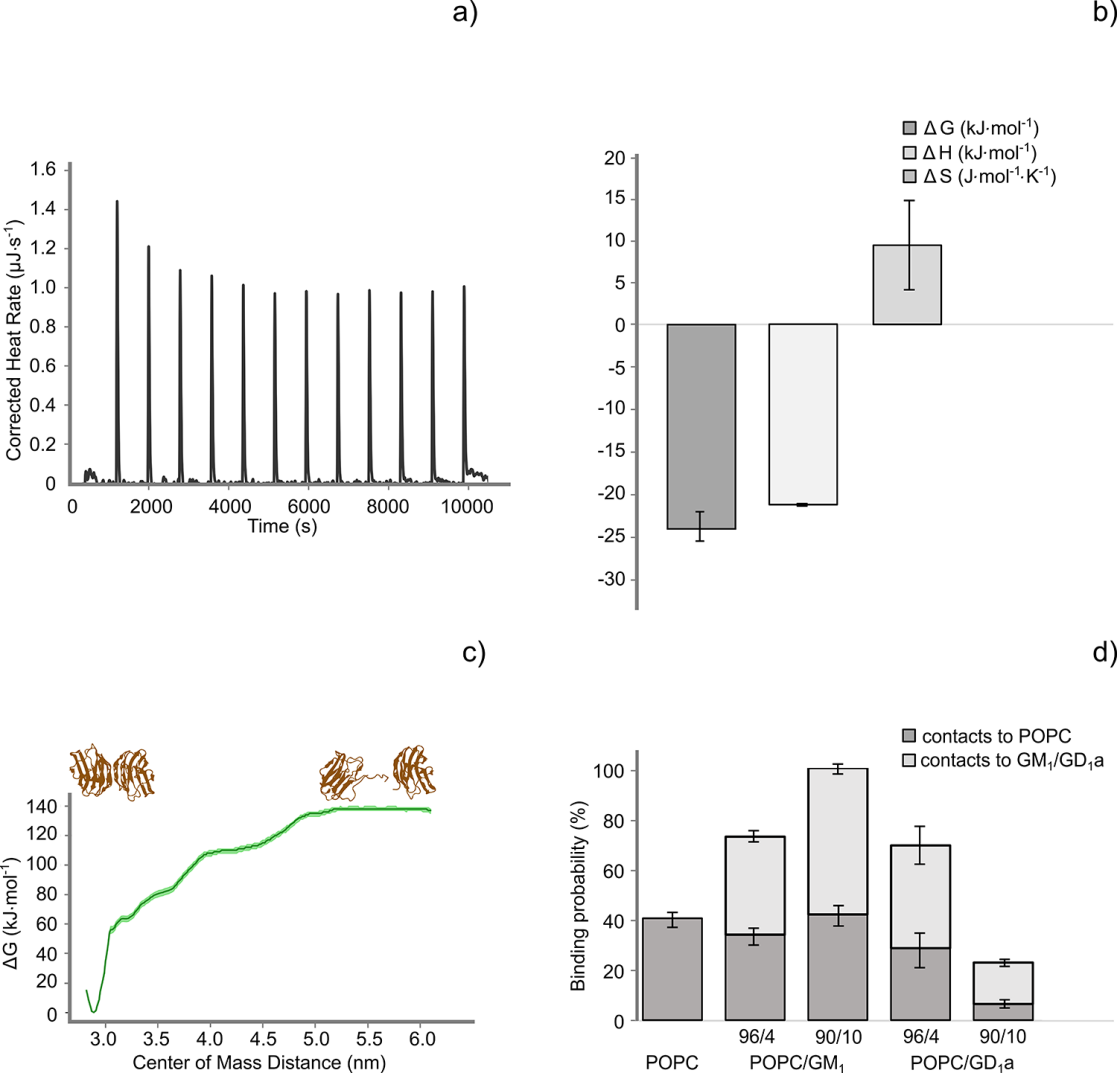
Thermodynamic study: ITC and all-atom MD simulations. (a) Representative thermogram depicting the corrected heat rate measured when adding 1.5 mM total lipid concentration of POPC/GM_1_ (96/4) to 2 μM of Gal-1 as a function of time. Controlled and precise additions of POPC/GM_1_ LUVs were performed to a solution of Gal-1 (10 mM PBS, 137 mM NaCl, and 0.27 mM KCl, pH = 7.4). Keeping the same instrumental setup, analogous additions to PBS (without Gal-1) of POPC/GM_1_ LUVs were made. These control experiments were necessary to obtain the blank titrations, which were subtracted from the sample measurements in the context of analyzing the reaction heats. (b) Thermodynamic parameters (Δ*G*, Δ*H*, Δ*S*) and their associated error bars indicating the standard deviation obtained titrating POPC/GM_1_ (96/4) to a Gal-1 solution. The thermodynamic data were gained from a global analysis performed by using HypCal software (*n* = 3). (c) PMF of Gal-1 dimer dissociation in water obtained using the umbrella sampling technique. The PMF was calculated using the weighted histogram analysis method implemented in GROMACS. Standard error bands (light green) are calculated using the Bayesian bootstrapping method. (d) Probability of the Gal-1 dimer binding to five different bilayers, i.e., POPC, POPC/GM_1_ (96/4), POPC/GM_1_ (90/10), POPC/GD_1_a (96/4), and POPC/GD_1_a (90/10), represented by the different bars. The bars’ dark and pale gray portions represent the contacts to POPC and GM_1_ or GD_1_a in each different bilayer composition, respectively (see Table 2 for details).

We employed HypCal software^68^ to determine thermodynamic parameters of the binding. Three curves were globally analyzed. We found an exothermic reaction with a related Δ*H* of (−21.39 ± 0.15) kJ mol^–1^ and a logK of 4.24 ± 0.30. Further analysis resulted in a small yet favorable entropic contribution Δ*S* of (9.4 ± 4.3) J K^–1^ mol^–1^, a Δ*G* of (−24.20 ± 1.7) kJ mol^–1^ (Figure 3b), and a *K*_d_ of (57 ± 17) μM. The latter value is about one magnitude higher than determined by QCM-D. The reason is likely because ITC detects the Gal-1 membrane interaction in bulk under defined concentrations and equilibrium conditions, while QCM-D characterizes the Gal-1 binding to an immobilized 2-dimensional membrane system with reproducible surface coverage by LUVs (see Table S1 in the Supporting Information). Although the enthalpy involved is of the same magnitude as the one reported for Gal-1 binding to bivalent disaccharides containing terminal galactose moieties, the entropies significantly differ.^69,70^ The entropy increase, related to the release of the water molecules from the Gal-1 CRD cavity upon binding to galactose of GM_1_ or bivalent disaccharides, is comparable.^71^ However, disaccharides diffuse in the solution,^69,70^ while GM_1_ is embedded in the lipid membrane, which gives a restricted number of starting configurations. Subsequently, a more constrained system undergoes a less significant loss of the degrees of freedom upon binding. On the other hand, the enthalpy is in the same range, pointing toward the involvement of the terminal galactose moiety of the GM_1_ in the interaction. The careful analysis of the ITC data suggests a 1:1 Gal-1:GM_1_ binding stoichiometry, which can be interpreted as an argument against the suggested GM_1_ cross-linking by the Gal-1 homodimer. That cross-linking is suggested to lead to the formation of a “glycan-galectin aggregation”.^72^ Although that concept has gained wide popularity, only very few studies show indirect proof of aggregate formation,^37^ specifically for the Gal-1 homodimer, a proof for GM_1_ cross-linking is missing.

In the context of GM_1_ cross-linking by Gal-1 it has to be considered that Gal-1 is mainly monomeric in solution at physiological concentrations.^73^ This finding agrees with the *K*_d_s reported in the literature in the micromolar range (2.5 ^74^ or 7 μM,^75–77^ respectively) and with our FCS experiments (see Supporting Information). Taking this into consideration, in the used concentration range from 2 to 10 μM, we expect to have between 80% and 40% of Gal-1 monomer in solution, depending on which K_d_ is used (for further details see Supporting Information and Figure S4). Similarly, some cell types express galectins at micromolar cytosolic concentrations.^73,78^ Thus, the majority of Gal-1 might be present as monomers not favoring cross-linking. However, cross-linking might still occur due to local accumulation of Gal-1 at the cell membrane upon specific cellular stimuli, leading to significantly higher local Gal-1 concentrations than used in our study (10 μM). Since galectins seem to possess more affinity for glycoproteins rather than glycolipids,^73^ Gal-1/glycoproteins binding could cause the increase of its local concentration, which might favor the subsequent binding of the Gal-1 homodimer to GM_1_. One might speculate that the elevated concentration of membrane-bound Gal-1 might in turn then lead to cross-linking, stabilizing the transient GM_1_ nanodomains,^58,59^ and by that the formation of stable “GM_1_/Gal-1 lattices”. At this point in the discussion, we shall mention further aspects reported in the literature relevant to the molecular mechanisms involved in that cross-linking: ligand binding to Gal-1 increases its affinity to form dimers,^79^ and a negative cooperativity was found for the carbohydrate binding to the dimeric form.^80,81^

All-atom MD simulations offer further insight into the stability of the Gal-1 homodimer and interactions of the homodimeric Gal-1 with different lipid species, which are not easily accessible by experiments. To address the formation of the Gal-1 homodimer, claimed to be a prerequisite for cross-linking of GM_1_ lipids or GM_1_-associated integrin,^27^ we assessed the stability of the Gal-1 homodimer in water by calculating the distance of the center of mass (COM) of the two monomers and by analyzing the secondary structure content (Figure S7). Figure S6 shows the time evolution of the COM distance between Gal-1 monomers in the homodimer. This distance remains constant, with an average of (2.89 ± 0.02) nm, suggesting that homodimeric Gal-1 is stable throughout the entire simulation (three repeats, each 1 μs long). To assess the changes in the secondary structure of the protein, we plot the time dependence of the secondary structure content for all repeats. As shown in Figure S7, there are no substantial changes in the secondary structure content of Gal-1 homodimer, again suggesting high dimer stability.

To gain further insight into the stability of the dimeric interface, we pulled Gal-1 monomers apart and calculated the free energy needed to dissociate the homodimer using the umbrella sampling technique.^52,53^ Figure 3c demonstrates that the homodimeric structure of Gal-1 is preferred and that the free energy difference between the dimeric and monomeric states is about 140 kJ/mol. Notably, the conditions in the MD simulations relate to a bulk concentration in the sub-mM range. Experiments yield *K*_d_s in the range of a few μM for the Gal-1 monomer–dimer equilibrium^74–77^ and thus confirm a stable homodimer at sub-mM concentrations, as shown in the simulations.

Subsequently, we explored the interactions of the Gal-1 homodimer with model lipid membranes by assessing the Gal-1 average interaction time and binding probability to different lipid species (Figures S8 and 3d, respectively). The Gal-1 dimer was initially placed about 2 nm above the bilayer surface and allowed to move freely in the simulation box. Five different lipid bilayer compositions have been used: POPC, POPC/GM_1_ (96/4), POPC/GM_1_ (90/10), POPC: (96/4), and POPC/GD_1_a (90/10) (see Table 2 for details). The binding probabilities of the Gal-1 dimer to different lipid species (shown in Figure 3d) have been calculated by counting the number of simulation frames where the Gal-1 dimer was in contact (within the distance of 0.6 nm) with the given lipid type. Our calculation unveiled intriguing patterns in the interaction dynamics between GM_1_ and the Gal-1 dimer. The binding probability experiences a notable upswing as the concentration of GM_1_ increases. Conversely, the probability of binding to GD_1_a exhibits a diminishing trend with increasing GD_1_a concentration. A comparative analysis with a pure POPC bilayer reveals that both POPC/GM_1_ and POPC/GD_1_a systems demonstrate somewhat elevated binding probabilities to the Gal-1 dimer (except for the system containing 10 mol % of GD_1_a, where overall Gal-1 homodimer binding is slightly smaller as compared to the pure POPC bilayer). It is essential to underscore that the binding probability to POPC is not negligible either, indicating the capacity of the Gal-1 dimer for nonspecific binding to lipid bilayers, even in the absence of ganglioside lipids. In summary, the trends observed in the MD simulations support our experimental view: Gal-1 binds specifically to GM_1_, but not to GD_1_a, and sub-mM concentrations of Gal-1 might favor the proposed GM_1_ cross-linking by Gal-1 homodimers.

## Conclusions

Synergistically with the binding to glycoproteins, the conversion of GD_1_a to GM_1_ supposedly triggers the binding of Gal-1 to the outer layer of the plasma membrane. This first concept is based on the elevated binding affinity of Gal-1 toward GM_1_ over GD_1_a when embedded in membranes, an assumption lacking experimental proof. In summary, our study unambiguously demonstrates that when probing a wide range of concentrations (i.e., 30 nM to 20 μM) Gal-1 binds indeed specifically to GM_1_, and not to GD_1_a when embedded in membranes at ganglioside concentrations of 4% with apparent dissociation constants of (4 ± 1) and (57 ± 17) μM for binding to immobilized and free-diffusing vesicles, respectively.

The second concept regarding the molecular mechanisms involved in the biological function of Gal-1 is the hypothesis of cross-linking of GM_1_’s by homobivalent Gal-1. The analysis of the ITC experiments indicates a 1:1 binding stoichiometry when using Gal-1 concentrations in the μM range. Considering that both the *K*_d_s for the Gal-1/GM_1_ interaction and for the Gal-1/Gal-1 dimerization^74–77^ are in the μM range, the investigated systems using μM concentrations must be in a dynamic equilibrium. Our results imply that forming such a “GM_1_/Gal-1 lattice”^72^ will certainly need higher than local micromolar Gal-1 concentrations. This calls for additional mechanisms driving both the Gal-1/Gal-1 dimerization and the GM_1_/Gal-1 equilibrium toward the membrane-bound homobivalent Gal-1. In vivo, this can be achieved by galectin accumulation in extracellular structures rich in galactoside residues, such as extracellular matrix or plasma membrane-proximal glycocalyx, as shown before.^38,82^ Similar trapping in glycostructures and deposition to surface receptors was described for secreted soluble signaling molecules, such as growth factors.^83^

## Supplementary Material



## Notes

Description of experimental procedures, characterization of Gal-1/TMR, description of dimeric/monomeric Gal-1 in equilibrium, validation of FRET and QCM-D methodology, and additional MD simulation data (PDF)
