# Supplementary material for: Unraveling the GM1 Specificity of Galectin‑1 Binding to Lipid Membranes
Source: ACS Bio Med Chem Au. 2025 May 7;5(3):415–26. doi: 10.1021/acsbiomedchemau.5c00040 (PMC12183518; doi:10.1021/acsbiomedchemau.5c00040)
Supplement: Supplementary file 1 [file bg5c00040_si_001.pdf]

Unraveling the GM_1 specificity of Galectin-1 binding to lipid membranes

Federica Scollo[1*], Waldemar Kulig[2], Gabriele Nicita[3], Anna-Kristin Ludwig[4], Joana C. Ricardo[1], Valeria Zito[5], Peter Kapusta[1], Ilpo Vattulainen[2], Marek Cebecauer[1], Hans-Joachim Gabius[4†], Herbert Kaltner[4], Giuseppe Maccarrone[3*], Martin Hof[1*]

[1]J. Heyrovský Institute of Physical Chemistry of the CAS, v. v. i., Dolejškova 2155/3, 182 23, Prague 8, Czech Republic

[2]Department of Physics, University of Helsinki, P.O. Box 64, FI-00014 Helsinki, Finland

[3]Dipartimento di Scienze Chimiche, Università degli Studi di Catania, Viale A. Doria 6,95125 Catania, Italy

[4] Ludwig-Maximilians-University Munich, Department of Veterinary Science, Chair of Biochemistry and Chemistry, Lena-Christ-Str.48, 82152 Planegg, Germany

[5]Istituto di Cristallografia, Consiglio Nazionale delle Ricerche, Via P. Gaifami 18, 95126, Catania, Italy

† Deceased August 2, 2021

* Corresponding authors ()

Email: federica.scollo@jh-inst.cas.cz, gmacca@unict.it, martin.hof@jh-inst.cas.cz

Supporting Information

TABLE OF CONTENT

Results and Discussion 3
Characterization of Gal-1/TMR 3
Choice of the FRET pair and calculation of the Förster Radius 3
Calculation of Degree of Labeling (DOL) 4
Fluorescence Correlation Spectroscopy characterization 5
Equilibrium dimer-monomer of Gal-1 6
Validation of the FRET methodology 8
Gal-1 QCM control and fitting of the data to determine the K_d 8
Stability of the Gal-1 homodimer 11
Interactions of Gal-1 dimer with lipid bilayers 12
References 13

Results and Discussion

Characterization of Gal-1/TMR

Choice of the FRET pair and calculation of the Förster Radius

The lifetime of Gal-1/TMR as a function of different concentrations of vesicles has been monitored to study their interaction. The labeled protein has been used as a FRET donor, whose fluorescence emission spectrum is reported in **fig. S1** (magenta curve). Considering the significant overlap with its UV-Vis spectrum (**fig. S1**, cyan curve), qualitatively highlighted in the **fig. S1** as a pale purple area, DOPE-Atto 633 (1% of the total lipid concentration) has been used as the FRET acceptor. This lipid conjugated dye is routinely employed in a large variety of fluorescence studies due to the feature of being easily intercalated into the lipid bilayer. [1]

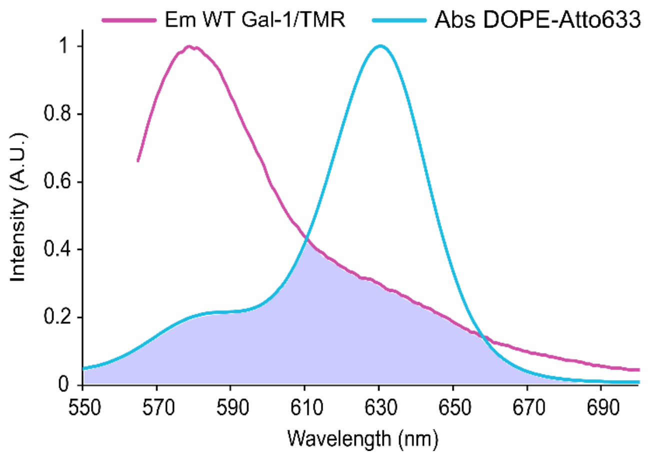

Figure S1. FRET pair used for the fluorescence measurements. The magenta spectrum shows the emission of Gal-1/TMR (**λ** $_{max}$ = 580 nm), whereas the light blue curve shows the absorption of the DOPE-Atto633 (**λ** $_{max}$ = 630 nm). In pale purple the spectral overlap between the two.

The Förster Radius of the above-described FRET pair was calculated by using [1]. [2]

$$R_0 = 0.211(\kappa^2 n^{-4} Q_D J_{(\lambda)})^{1/6} \quad [1]$$

Where κ^2 is a factor considering the relative orientation of the FRET pair's transition dipoles, usually approximated to 2/3, [2] n is the refractive index of the solvent and Q_D is the quantum yield of the donor. $J_{(\lambda)}$is the overlap integrand and it is defined as:

$$J_{(\lambda)} = \int_0^{\infty} F_{D(\lambda)} \varepsilon_{A(\lambda)} \lambda^4 d\lambda \quad [2]$$

Where $F_{D\,(\lambda)}$ is the corrected fluorescence intensity of the donor with the total intensity, between λ and $\lambda + \Delta\lambda$, and the $\varepsilon_{A(\lambda)}$ is the extinction coefficient of the acceptor as a function of λ. For the FRET pair used in our study, (i.e. TMR and Atto-633 respectively conjugated to the protein and the lipid in the membrane, **fig.S1**) we determined a Förster radius of 45 Å.

Calculation of Degree of Labeling (DOL)

Gal-1 was labelled with TMR-maleimide following the procedure reported in **Experimental Procedures**. TMR-maleimide has been chosen because it selectively reacts via cysteine, also preventing the protein oxidation which would affect its activity. The Gal-1 possesses six cysteines per monomer, i.e., Cys2, Cys16, Cys42, Cys60, Cys88 and Cys130, two of them (C2 and C135) are the most exposed to the solvent, [3] thus the most reactive, and also the one responsible for the tertiary and quaternary structures. [4] The DOL is defined as the ratio between the moles of the dye per moles of Gal-1, and it was calculated using UV-Vis spectroscopy. The spectrum was acquired at room temperature using a Shimadzu Europe UV-2600 spectrophotometer in the range of 200–700 nm. Briefly, the concentration of TMR was measured by monitoring the absorbance at 552 nm and the concentration of Gal-1/TMR considering the absorbance at 280 nm, to which the contribution of the dye has been subtracted, knowing from other experiments the extinction coefficient of the dye at 280 nm and the respective concentrations (**fig. S2**).
The UV-Vis spectrum at 280 nm is attributed to two contributions, i.e., the TMR and the Gal-1, as expressed in the **eq. 3**.

$$A_{280} = A_{280\ TMR} + A_{280\ Gal-1} [3]$$

$$A_{280} = C_{TMR}\varepsilon_{280\,TMR} + C_{Gal-1}\ \varepsilon_{280\ Gal-1} [4]$$

$$C_{Gal-1} = \frac{A_{280} - C_{TMR}\varepsilon_{280\ TMR}}{\varepsilon_{280\ Gal-1}} [5]$$

where C is the concentration, A is the measured absorbance and ε is the molar absorptivity in M^{-1} cm^{-1}. The concentration of the dye (C_{TMR}) was obtained using the A_{552nm} of the measured spectrum (**Fig. S3A**) due to the TMR only. The ε_{552} was calculated by measuring three UV-vis spectra at three different concentrations of the TMR-maleimide in mQ water. The slopes of the three different linear curves are the ε at 552 nm, 515 nm and 280 nm (**Fig S2**).

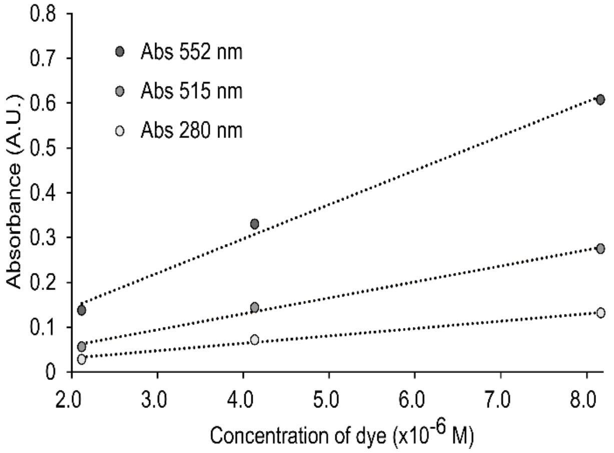

Fig. S2. Absorbance of TMR-maleimide in mQ water as a function of the concentration. Three different wavelengths have been plotted. The R-square are 0.9927, 0.9042, and 0.9884, respectively, for dark grey, grey and light grey curves.

Gal-1's $\varepsilon^{1\%}_{1\,cm}$ is 5.4, as stated elsewhere. [5] We used this value upon conversion into the molar absorptivity expressed in $mol^{-1}cm^{-1}$. Using the **eq. 3**, the DOL resulted in 0.6.

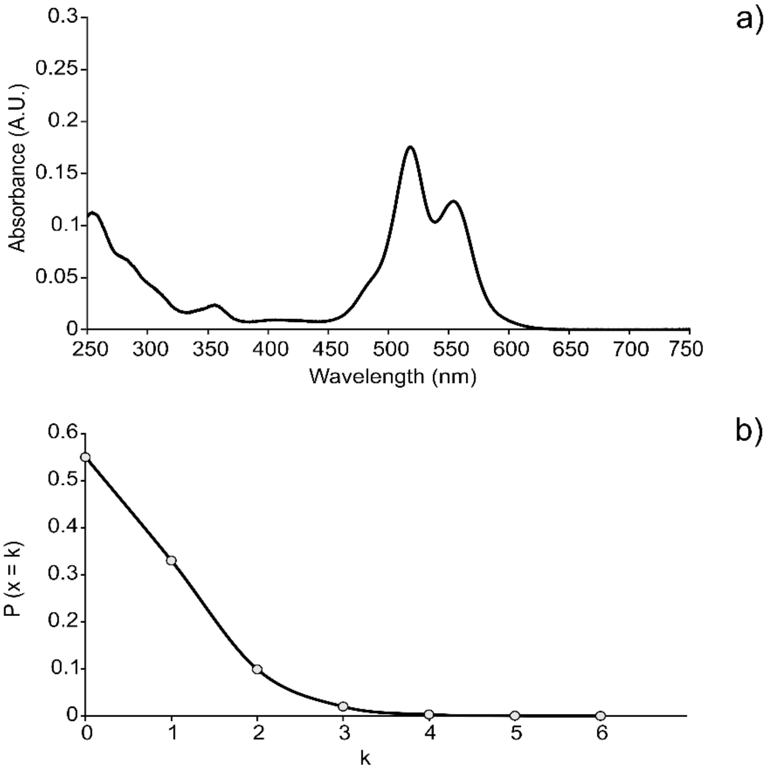

Figure S3. A) UV-Vis spectrum of Gal-1/TMR. B) Poisson distribution to the corresponding DOL 0.6.

From a photophysical point of view, TMR dye is known for forming of H-dimers under certain conditions. [6-8] Gal-1/TMR is characterized by a significant amount of H-dimers, as shown by the UV-vis spectra reported in **Fig. S3a**. Since the characteristic absorption spectrum of TMR shows a unique maximum at around 552 nm and only a barely visible shoulder at around 520 nm, the pronounced maximum at around 520 nm of the Gal-1/TMR indicates that many Gal-1 molecules carry more than one TMR molecule. [7] The Poisson distribution corresponding to a DOL of 0.6 is reported in **Figure S3b**. The distribution shows that there is roughly 55% of the protein which did not react with the dye, thus unlabeled, 33% of the population is represented by the 1:1 (protein to dye) species, 10% by the 1:2 species, and 2% of the protein is labelled with 3 dyes. While these features do not shatter the soundness of the FRET titrations (**Fig. 1c, d, e, f**) since the control is always measured, on the other hand, it might be one of the reasons preventing us from obtaining the classical binding curve trend.

Fluorescence Correlation Spectroscopy characterization

To characterize Gal-1/TMR we employed FCS. We acquired single-point measurements in solution, each of them for 2 min. The global analysis of multiple measurements was performed using SymPhoTime 64 Software. The autocorrelation curves were fitted keeping the same boundaries (0.002-1000 ms), choosing a 3D free diffusion model, single species described in the eq. [6] as follows:

$$G_{(t)} = \frac{1}{N}\frac{1}{1+\left(\frac{t}{\tau}\right)}\sqrt{\left(\frac{1}{1+\left(\frac{t}{\tau\kappa^{2}}\right)}\right)} \quad [6]$$

where N is the number of independently diffusing species within the confocal volume, τ is the mean diffusion time, and κ is the structural parameter describing the shape of the confocal volume. [9-12] Size of the confocal volume and κ were calculated for aqueous solution of rhodamine-B or the Alexa-532 as a standard (D = 427 ± 4 μm^2/s and 396 ± 10 μm^2/s at 298.15 K and 295.65 K, respectively). [13, 14] The mean diffusion time of the free Gal-1/TMR determined this way was τ = 0.138 ± 0.003 ms, and from that we calculated the diffusion coefficient (D) using the eq. [7]

$$D = \frac{{\omega_0}^2}{4\tau} \quad [7]$$

where ω_0 is the waist of the confocal volume calculated using rhodamine-B or Alexa-532, as described above. The volume of the confocal volume was (0.77 ± 0.04) fL. This procedure resulted in a D = 135 ± 2 μm^2·s^{-1} (n=26). From the comparison with the values for the diffusion constants reported in [15] of Gal-1 monomer and homodimer (i.e., 130 and 105 μm^2·s^{-1}, respectively), we conclude that Gal-1/TMR is a monomer at 50-100 nM which is in line with literature reported equilibrium constants. [16-19]

Equilibrium dimer-monomer of Gal-1

To evaluate the concentration of dimeric and monomeric species present in solution at different protein concentrations adopted in this study, the equilibrium [8] and the mass balance equation [10] were used.

$$GG \rightleftharpoons G + G \quad [8]$$

Where GG is the Gal-1 homodimer and G is the Gal-1 in the monomeric form. This equilibrium is associated with a dimerization constant (K) as follows:

$$K = \frac{[G]^2}{[GG]} \quad [9]$$

Finally, according to the mass balance law:

$$C_{(Gal-1)} = [G] + [GG] \quad [10]$$

Where $C_{(Gal-1)}$ is the analytical concentration of the protein. Using eq. [9] and [10], we obtain:

$$[G]^2 + K[G] - KC_{Gal-1} = 0 \quad [11]$$

We used the obtained equation [11] to write a python script. The script enabled us to obtain a distribution diagram for the two species present in the equilibrium under study under the wide concentration range utilized in this work (**Fig. S4**). The script only requires the dimerization constants [16-19] and the concentration range as inputs.

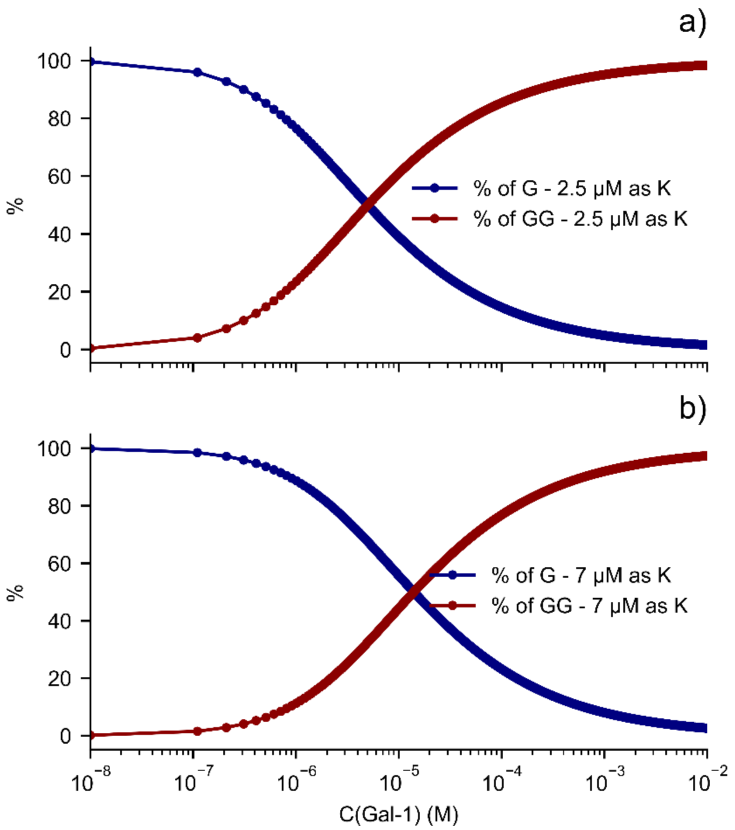

Figure S4. Distribution diagram representing the percentage of Gal-1 monomer (G, blue curve) and Gal-1 homodimer (GG, red curve) as a function of Gal-1 concentration utilizing A) a dimerization constant of 2.5 µM [19] and B) a dimerization constant of 7 µM.[16-18]

Validation of the FRET methodology

To further validate our FRET methodology reported in the main text (**Fig. 1c, d, e, f**), we took advantage of the high affinity of cholera toxin for GM_1, which has been extensively studied and reported in previous works. [20-22] Cholera toxin has been chosen as a positive control to study the binding to POPC:GM_1 vesicles, as previously done for Gal-1 and reported in **Fig. 1c, d, e,** and **f**. We performed the same qualitative assay employing commercially available cholera toxin-Alexa488. Its lifetime was monitored as a function of total phospholipids concentration for three different LUVs composition, i.e. POPC (+1% of DOPE-Rhod), POPC:GM_1 (+1% of DOPE-Rhod) and POPC:GM_1 without the acceptor. The data are reported in **fig. S4** and clearly show that the effect of the decreasing lifetime as a consequence of the energy transfer can be detected and is not due to any other experimental artifact.

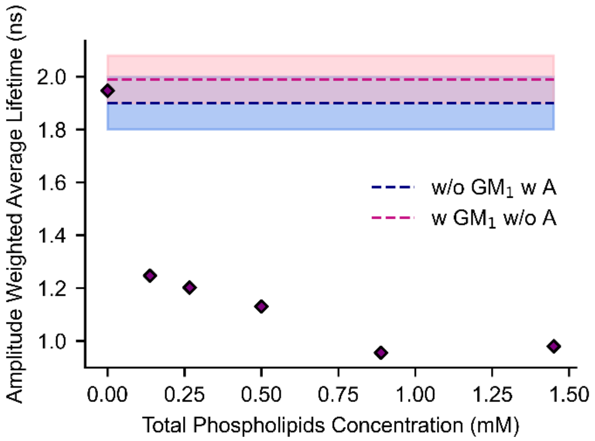

Figure S5. Amplitude weighted average lifetime of the cholera toxin-Alexa488 (500 nM) as a function of total phospholipids concentration. Three different compositions were measured. The average of the lifetimes was plotted as a dashed blue line for POPC (+ 1% of DOPE-Rhod as acceptor, named A in the graph). The average of the lifetimes was plotted as a dashed magenta line for POPC:GM_1 without acceptor. For POPC:GM_1 (96/4) (+1% of DOPE-Rhod) the lifetime was plotted against the concentration of lipid vesicles as purple rhombs.

Gal-1 QCM control and fitting of the data to determine the K_d

We employed QCM-D to both qualitatively show the specificity of Gal-1 for GM_1 containing membrane and quantitatively determine the apparent K_d of the binding to POPC:GM_1 LUVs deposited onto the gold sensor (**Fig. 2a, c** and **d**). The flow of the experiment is represented in **Fig. 2b**. Briefly, each independent experiment consisted of three steps after treating the sensor, measuring the frequency in air and in the PBS buffer (PBS 10 mM, NaCl 157 mM, KCl 0.27 mM, pH=7.4): I) Injection of the desired amount of vesicles (in huge excess compared to the available surface of the sensor) until stabilization of the frequency, followed by buffer perfusion; II) Injection of inert lipid composition, i.e., POPC, again followed by buffer. This step was introduced to reach the maximum coverage of the sensor and prevent its interaction with the protein; III) perfusion of the desired amount of Gal-1, followed by buffer perfusion after stabilization of the frequency. The frequency shift (Δf) was calculated for each step after the related buffer injection. The washing step with the buffer ensures the exclusion of all the material which is previously deposited of the sensor in a-specific manner. The sensor was treated as described elsewhere [23] so that the pre-extruded vesicles would physically adsorb onto the gold sensor without bursting, therefore without forming supported lipid bilayers. However, due to geometric and sterically constrained, this method renders the complete coverage of the sensor a difficult task to achieve. The first experimental precaution was to carry out the second step when injecting compositions different from POPC. The vesicles deposition is rather a stochastic phenomenon,

not only based on gravity and diffusion laws, but also affected by electrostatic interactions between vesicles themselves, and with the sensor. The matter complicates when thinking that the size of the vesicles is different and it is a distribution of sizes. All these factors render the maximum frequency variation obtained by injecting vesicles of various compositions different. However, a high degree of coverage is desirable to avoid interferences coming from the direct interaction of the Gal-1 with the naked sensor, especially considering that Gal-1 possesses six cysteine residues per monomer. The derived frequency shift would be summed to the eventual frequency shift caused by the direct interaction with the lipid membranes and the two contributions cannot be separated nor distinguished. This possible artifact would bias the binding detection and the subsequent K_d determination. To exclude this scenario in the context of the determination of the binding constant (POPC:GM_1 only, **Fig. 2d**), we replicated the experiment three times for each concentration, and we focused on the possible correlation between Δf due to the different steps (I, II and III – **Fig. 2b**). The Δf were obtained by subtracting the frequency before the injection to the frequency reached after the washing step performed after each step. The Δf related to the three different steps are reported in **Table S1**.

Table S1. Frequency shifts (Δf) of the three different steps in the QCM-D experiments on POPC:GM_1 (96/4).

Gal-1 Concentration (μM)	Δf (I step) (Hz)	Δf (II step) (Hz)	Δf (III step) (Hz)	Sum of Δf (I and II steps) (Hz)
0.75	211	13	1	224
	215	17	7	232
	204	11	8	215
1.25	244	4	9	248
	227	17	13	244
	**191**	**6**	**5**	**197**
2.50	258	4	13	262
	198	10	12	208
	246	17	17	263
5.00	226	17	22	243
	262	7	19	269
	**263**	**10**	**16**	**273**
10.00	238	18	24	256
	209	22	25	231
	206	12	28	218

The sum of the I and the II steps is directly proportional to the quantity of vesicles deposited onto the sensor prior to the perfusion of Gal-1. As they oscillate between 197 Hz and 273 Hz (bold lines in the table), these values feature a high variability, likely due to differences in the size distribution of the extruded vesicles (~120 nm, measured by Dynamic Light Scattering—data not shown). To help the reader visualize the data, the values were categorized into three distinct groups based on their distribution:(i) a first group centered around 210 Hz, (ii) a second group centered around 235 Hz, and (iii) a third group centered around 260 Hz. If the highest amount of Gal-1, thus the highest frequency shift, was not due to the binding of the protein with the vesicles but to nonspecific interaction with the gold sensor, the amount of Gal-1 detected on the sensor (with pre-deposited vesicles) would be inversely proportional to the degree of coverage, i.e., the sum of the frequency shifts of the I and the II steps. In other words, if fewer vesicles were present on the sensor, the frequency shift would be lower, and a greater exposed sensor area should lead to increased Gal-1 detection during the third step. However, this is clearly not the case, as evidenced by the data in the table, both in terms of overall trends and within the replicates at the same protein concentration.

This modification of the protocol was needed to avoid potential artifacts stemming from the direct interaction between the protein and the gold sensor. Indeed, this was necessary to differentiate between the two contributions to the frequency shift, i.e., the protein interacting with the lipid vesicles and/or with the sensor (see **S.I., Table 1** to see the data related to step I and II, i.e., vesicles deposition). A schematic drawing of the experiment's flow is depicted in **Fig. 2b**. The data reported in **Table 1** and represented in **Fig. 2d** were used to determine the dissociation constant of the Gal-1 binding to POPC:GM_1 (96/4) vesicle deposited onto the sensor. The frequency shifts observed upon protein injection (III step) were plotted against the concentrations of Gal-1 used. Those data were fitted using the equation reported in previous work. [24]

Stability of the Gal-1 homodimer

To assess the stability of the Gal-1 homodimer in the MD simulations, the center of mass distance between the Gal-1 monomers in solution has been plotted in **Fig. S6**. Data clearly show that the homodimer is very stable, with an average distance of (2.89 ± 0.02) nm between the monomers.

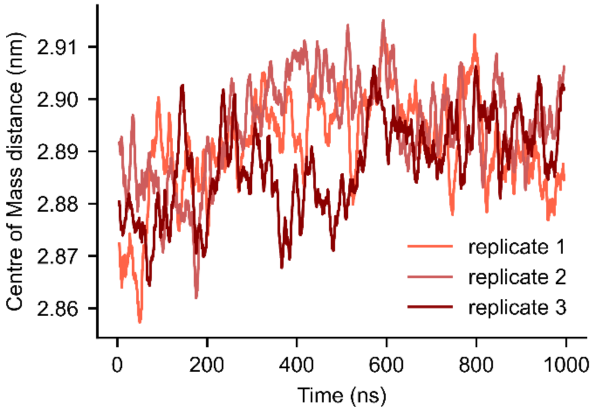

Figure S6. Time-dependence of the center of mass distance between Gal-1 monomeric units in the Gal-1 homodimer in solution obtained from the all-atom MD simulations. Data collected from three independent simulations plotted in the three different shades of red.

Additionally, the secondary structure content has been analyzed and depicted in **Fig. S7**. No substantial changes in the secondary structure content are observed suggesting high stability of the Gal-1 homodimer.

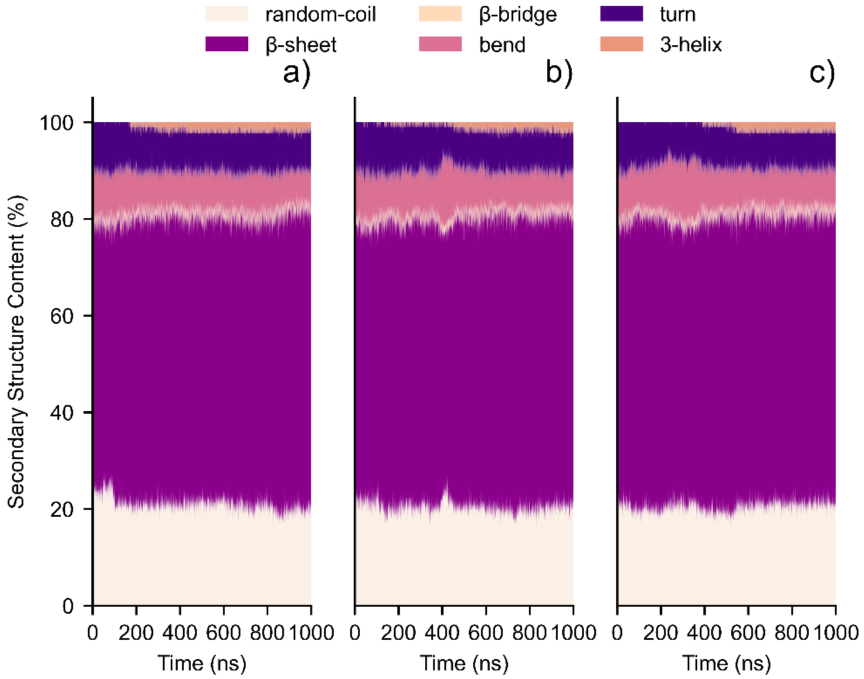

Figure S7. Time-dependence of the secondary structure content of the Gal-1 homodimer in solution. Each panel presents the secondary structure content from a single MD simulation. Different types of the secondary structures are color-coded as follows: random coil – linen gray, beta-sheet – dark magenta, beta-bridge - peach, bend – pale violet, turn - purple and 3-helix - dark salmon.

Interactions of Gal-1 dimer with lipid bilayers

In addition to binding probabilities showed in the manuscript (see **Fig. 3d**), we calculated the average interaction times between the Gal-1 homodimer and lipid membrane depicted in **Fig. S8**. The average interaction times and standard deviations were calculated from the decay of the time auto-correlation functions by identifying the times when the time auto-correlation function decreased to zero. Results depicted in **Fig. S8** clearly indicate that the average interaction times between the Gal-1 dimer and lipid membrane containing GM_1 are higher as compared to the interactions with lipid bilayer containing GD_1a.

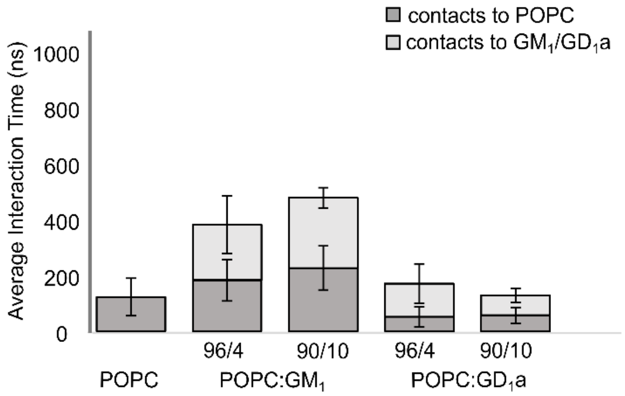

Figure S8. Average times and associated standard deviations of the interaction between Gal-1 homodimer binding to five different bilayers, i.e., POPC, POPC:GM_1 (96/4), POPC:GM_1 (90/10), POPC:GD_1a (96/4), POPC:GD_1a (90/10), represented by the different bars. The bars' dark and pale grey portions represent the contacts to POPC and GM_1 or GD_1a in each different bilayer composition, respectively (see **Table S2** for details).

References

1. Kulakowska, A.; Jurkiewicz, P.; Sykora, J.; Benda, A.; Mely, Y.; Hof, M., Fluorescence Lifetime Tuning-A Novel Approach to Study Flip-Flop Kinetics in Supported Phospholipid Bilayers. *Journal of Fluorescence* **2010,** *20*, 563-569.
2. Lakowicz, J. R., *Principles of Fluorescence Spectroscopy*. 3rd ed.; 2006.
3. Guardia, C. M. A.; Gauto, D. F.; Di Lella, S.; Rabinovich, G. A.; Martí, M. A.; Estrin, D. A., An Integrated Computational Analysis of the Structure, Dynamics, and Ligand Binding Interactions of the Human Galectin Network. *J. Chem Inf. Model.* **2011,** *51* (8), 1918-1930.
4. Tracey, B. M.; Feizi, T.; Abbott, W. M.; Carruthers, R. A.; Green, B. N.; Lawson, A. M., Subunit molecular mass assignment of 14,654 Da to the soluble beta-galactoside-binding lectin from bovine heart muscle and demonstration of intramolecular disulfide bonding associated with oxidative inactivation. *Journal of Biological Chemistry* **1992,** *267* (15), 10342-10347.
5. Ahmad, N.; Gabius, H. J.; Sabesan, S.; Oscarson, S.; Brewer, C. F., Thermodynamic binding studies of bivalent oligosaccharides to galectin-1, galectin-3, and the carbohydrate recognition domain of galectin-3. *Glycobiology* **2004,** *14* (9), 817-825.
6. Diaz-Garcia, C.; Renart, M. L.; Poveda, J. A.; Giudici, A. M.; Gonzalez-Ros, J. M.; Prieto, M.; Coutinho, A., Probing the Structural Dynamics of the Activation Gate of KcsA Using Homo-FRET Measurements. *Int. J. Mol. Sci.* **2021,** *22* (21), 21.
7. Ogawa, M.; Kosaka, N.; Choyke, P. L.; Kobayashi, H., H-Type Dimer Formation of Fluorophores: A Mechanism for Activatable, *in Vivo* Optical Molecular Imaging. *ACS Chem. Biol.* **2009,** *4* (7), 535-546.
8. Donaphon, B.; Bloom, L. B.; Levitus, M., Photophysical characterization of interchromophoric interactions between rhodamine dyes conjugated to proteins. *Methods Appl. Fluoresc.* **2018,** *6* (4).
9. Magde, D.; Elson, E. L.; Webb, W. W., Fluorescence correlation spectroscopy. II. An experimental realization. *Biopolymers* **1974,** *13* (1), 29-61.
10. Elson, E. L.; Magde, D., Fluorescence correlation spectroscopy. I. Conceptual basis and theory. *Biopolymers* **1974,** *13* (1), 1-27.
11. Aragon, S. R.; Pecora, R., Fluorescence correlation spectroscopy as a probe of molecular dynamics. *Journal of Chemical Physics* **1976,** *64* (4), 1791-1803.
12. Machan, R.; Jurkiewicz, P.; Olzynska, A.; Olsinova, M.; Cebecauer, M.; Marquette, A.; Bechinger, B.; Hof, M., Peripheral and Integral Membrane Binding of Peptides Characterized by Time-Dependent Fluorescence Shifts: Focus on Antimicrobial Peptide LAH(4). *Langmuir* **2014,** *30* (21), 6171-6179.
13. Kapusta, P., Absolute diffusion coefficients: compilation of reference data for FCS calibration. *PicoQuant GmbH* **2010,** *1*, 1-2.
14. Tressler, C.; Stolle, M.; Fradin, C., Fluorescence correlation spectroscopy with a doughnut-shaped excitation profile as a characterization tool in STED microscopy. *Opt. Express* **2014,** *22* (25), 31154-31166.
15. Göhler, A. Untersuchung Karbohydrat-bindender Proteine mit hoher zeitlicher und räumlicher Auflösung. University Wuerzburg, **2012**.
16. Cho, M.; Cummings, R. D., Characterization of monomeric forms of galectin-1 generated by site-directed mutagenesis. *Biochemistry* **1996,** *35* (40), 13081-13088.
17. Cho, M. J.; Cummings, R. D., Galectin-1, a beta-galactoside-binding lectin in chinese-hamster ovary cells .2. Localization and biosynthesis. *J. Biol. Chem.* **1995,** *270* (10), 5207-5212.
18. Cho, M. J.; Cummings, R. D., Galectin-1, a beta-galactoside-binding lectin in chinese-hamster ovary cells .1. Physical and chemical characterization. *J. Biol. Chem.* **1995,** *270* (10), 5198-5206.
19. Salomonsson, E.; Larumbe, A.; Tejler, J.; Tullberg, E.; Rydberg, H.; Sundin, A.; Khabut, A.; Frejd, T.; Lobsanov, Y. D.; Rini, J. M.; Nilsson, U. J.; Leffler, H., Monovalent Interactions of Galectin-1. *Biochemistry* **2010,** *49* (44), 9518-9532.
20. Kuziemko, G. M.; Stroh, M.; Stevens, R. C., Cholera toxin binding affinity and specificity for gangliosides determined by surface plasmon resonance. *Biochemistry* **1996,** *35* (20), 6375-6384.

21. Cuatrecasas, P., Interaction of *Vibrio cholerae* Enterotoxin with Cell Membranes. *Biochemistry* **1973,** *12* (18), 3547-3558.
22. Holmgren, J.; Lonnroth, I.; Svennerholm, L., Tissue Receptor for Cholera Exotoxin: Postulated Structure from Studies with GM_1 Ganglioside and Related Glycolipids. *Infection and Immunity* **1973,** *8* (2), 208-214.
23. Keller, C. A.; Kasemo, B., Surface specific kinetics of lipid vesicle adsorption measured with a quartz crystal microbalance. *Biophys J* **1998,** *75* (3), 1397-1402.
24. Su, Q.; Vogt, S.; Nöll, G., Langmuir Analysis of the Binding Affinity and Kinetics for Surface Tethered Duplex DNA and a Ligand-Apoprotein Complex. *Langmuir* **2018,** *34* (49), 14738-14748.
